# fMRI Resting Slow Fluctuations Correlate with the Activity of Fast Cortico-Cortical Physiological Connections

**DOI:** 10.1371/journal.pone.0052660

**Published:** 2012-12-20

**Authors:** Giacomo Koch, Marco Bozzali, Sonia Bonnì, Viola Giacobbe, Carlo Caltagirone, Mara Cercignani

**Affiliations:** 1 Laboratory of Clinical and Behavioural Neurology, Santa Lucia Foundation IRCCS, Rome, Italy; 2 Department of Neuroscience, University of Rome Tor Vergata, Rome, Italy; 3 Neuroimaging Laboratory, Santa Lucia Foundation IRCCS, Rome, Italy; 4 Clinical Imaging Sciences Centre, Brighton & Sussex Medical School, University of Sussex, Falmer, England, United Kingdom; Beijing Normal University, Beijing, China

## Abstract

Recording of slow spontaneous fluctuations at rest using functional magnetic resonance imaging (fMRI) allows distinct long-range cortical networks to be identified. The neuronal basis of connectivity as assessed by resting-state fMRI still needs to be fully clarified, considering that these signals are an indirect measure of neuronal activity, reflecting slow local variations in de-oxyhaemoglobin concentration. Here, we combined fMRI with multifocal transcranial magnetic stimulation (TMS), a technique that allows the investigation of the causal neurophysiological interactions occurring in specific cortico-cortical connections. We investigated whether the physiological properties of parieto-frontal circuits mapped with short-latency multifocal TMS at rest may have some relationship with the resting-state fMRI measures of specific resting-state functional networks (RSNs). Results showed that the activity of fast cortico-cortical physiological interactions occurring in the millisecond range correlated selectively with the coupling of fMRI slow oscillations within the same cortical areas that form part of the dorsal attention network, i.e., the attention system believed to be involved in reorientation of attention. We conclude that resting-state fMRI ongoing slow fluctuations likely reflect the interaction of underlying physiological cortico-cortical connections.

## Introduction

In the human brain complex networks rather than isolated cortical areas sub-serve specific brain functions, such as, for instance, movement, memory, or attention [1]. Notably, this anatomo-functional organization emerges not only when the brain is engaged in performing tasks, but also when neural activity is recorded at rest [2], [3]. Using functional magnetic resonance imaging (fMRI), recordings of spontaneous fluctuations of the blood-oxygenation level dependent (BOLD) signal at rest have produced consistent results across studies, by returning well reproducible resting-state functional networks (RSNs) [2], [3], [4]. To date, several functional RSNs have been identified and associated to specific brain functions, including for instance motor functions, visual and auditory processing, attention, and global cognition [5], [6].

In humans, simultaneous recording of electroencephalography (EEG) and fMRI at rest has returned significant correlations between the variations of band power of different cortical rhythms and BOLD fluctuations within specific brain networks [7], [8]. Moreover, it was recently demonstrated that resting-state fMRI reflects certain aspects of space oriented EEG analysis, with millisecond time resolution [9], [10]. Nevertheless, the relationship between the fluctuations of BOLD signal and neuronal activity of interconnected networks still remains controversial [11], especially considering that the BOLD signal is an indirect measure of neuronal activity, reflecting slow local variations in de-oxyhaemoglobin concentration [12]. In particular, it remains to be demonstrated that measures of the level of synchronization in the BOLD signal between different brain areas quantifies the actual degree of causal physiological connectivity between the same areas.

A unique opportunity to challenge the neurophysiological characteristics of functional connectivity of the brain at rest is provided by multifocal transcranial magnetic stimulation (TMS) [13], [14]. Using this method, a conditioning TMS pulse can be applied over a target cortical area (i.e., the posterior parietal cortex-PPC) shortly before a test pulse is delivered over the hand area of the primary motor cortex (M1). At appropriate interstimulus intervals, the motor evoked response (MEP) elicited by M1 stimulation is modulated, indicating the presence of functional interactions between the two sites (PPC and M1) [15]. This approach can also be extended to a third cortical site, by applying an additional TMS stimulus over another crucial node of the network (trifocal TMS). This latter pulse can be used to test how a third cortical area can impact on the functional connectivity assessed by bifocal TMS [16].

Here, we aimed to investigate whether the physiological properties of these circuits may have a relationship with the resting-state fMRI measures, hypothesizing that BOLD fluctuations reflect underlying neurophysiological interactions. Therefore, we used correlation analyses to verify whether, across subjects, specific associations exist between connectivity in parieto-frontal circuits assessed by multifocal TMS, and BOLD signal changes in well-known RSNs whose nodes overlap with the sites of TMS stimulation.

## Materials and Methods

### Participants

In total, 19 healthy volunteers (7 males and 12 females; age ranging from 21 to 36 years) took part in this study. The participants are a subsample of those enrolled for a previous study, in which we combined TMS and diffusion imaging data [16]. All subjects had to be right-handed based on the Edinburgh Handedness Inventory [17]. The experimental procedures used here were approved by the local Ethics Committee of Santa Lucia Foundation (Rome, Italy) and were carried out in accordance with the Declaration of Helsinki. Written informed consent was obtained from all recruited subjects before study initiation. MRI acquisition and TMS recordings were performed in two consecutive days for every subject. Subjects refrained from caffeine, nicotine and alcohol prior to the recordings, and all experiments were run at the same daytime.

### MRI Acquisition

All subjects underwent an MRI examination, obtained at 3 T (Magnetom Allegra, Siemens, Erlangen, Germany), including the following sequences: (1) dual echo turbo spin echo (TR:6190 ms, TE:12/109 ms, matrix:2563192, FOV:230×172.5, 48 contiguous slices, slice thickness:3 mm, total scan time:4 min); (2) three-dimensional modified driven equilibrium Fourier transform (MDEFT) scan (TR:1338 ms, TE:2.4 ms, TI:910 ms, flip angle:15°, matrix:256×224×176, in plane FOV:256×224 mm^2^, slice thickness:1 mm, total scan time:12 min); (3) T2* weighted echo planar imaging (EPI) sensitised to blood oxygenation level dependent imaging (BOLD) contrast (TR:2080 ms, TE:30 ms, 32 axial slices parallel to AC-PC line, matrix:64×64, pixel size:3×3 mm^2^, slice thickness:2.5 mm, flip angle:70°) for resting state fMRI. BOLD echo planar images were collected during rest for a 7 min and 20 s period, resulting in a total of 220 volumes. During this acquisition, subjects were instructed to keep their eyes closed, not to think of anything in particular, and not to fall asleep.

### Resting state fMRI Analysis

Data were pre-processed using Statistical Parametric Mapping (Wellcome Department of Imaging Neuroscience; SPM8), and in-house software implemented in Matlab (The Mathworks Inc, Natick, Massachussetts, USA). For each subject, the first four volumes of the fMRI series were discarded to allow for T1 equilibration effects. The pre-processing steps included correction for head motion, compensation for slice-dependent time shifts, normalization to the EPI template in MNI coordinates provided with SPM8, and smoothing with a 3D Gaussian Kernel with 8mm^3^ full-width at half maximum. Then, all images were filtered by a phase-insensitive band-pass filter (pass band 0.01-0.08 Hz) to reduce the effect of low frequency drift and high frequency physiological noise.

We employed independent component analysis (ICA) (group ICA for fMRI toolbox, GIFT, http://icatb.sourceforge.net/) to identify, on a subject by subject basis, regions belonging to previously identified resting-state networks (RSNs). ICA is a data-driven approach to extract independently distributed spatial patterns from fMRI data. In simple words, ICA unmixes a number of spatial “sources” from the whole data set, identifying the temporal pattern associated with each of them. In the case of resting-state fMRI the sources correspond to spatially independent brain regions, each associated with a specific time-course. ICA analysis was set to identify 20 independent components. Briefly, GIFT first concatenates the individual data across time, and then produces a computation of subject specific components and time courses. For all subjects grouped together, the toolbox performed the analysis in three steps: 1) data reduction, 2) application of the FastICA algorithm, and 3) back-reconstruction for each individual subject [18]. Results were converted to Z-scores in order to perform parametric statistics on these images. The 20 components were reviewed, discarding those corresponding to brain tissue compartments of no interest (i.e., white matter and cerebrospinal fluid) and to artefacts. Among the remaining components, we identified seven spatial patterns corresponding to RSNs which have been consistently recognised by other groups [8], [19]. A detailed description is given in the results section. Given the sites used for TMS stimulation (see next section), and in line with our hypothesis, we retained only the RSNs whose nodes include at least some of the stimulation sites. These are: the default mode network (DMN), which includes the angular gyrus bilaterally; the dorsal attention network (DAN), which includes the intra-parietal sulcus and the middle frontal gyrus (premotor cortex) bilaterally; and the sensory-motor network (SMN), which includes M1. In order to minimize the number of statistical comparisons, we restricted our analysis to these 3 networks.

### TMS recordings

**Figure 1:**
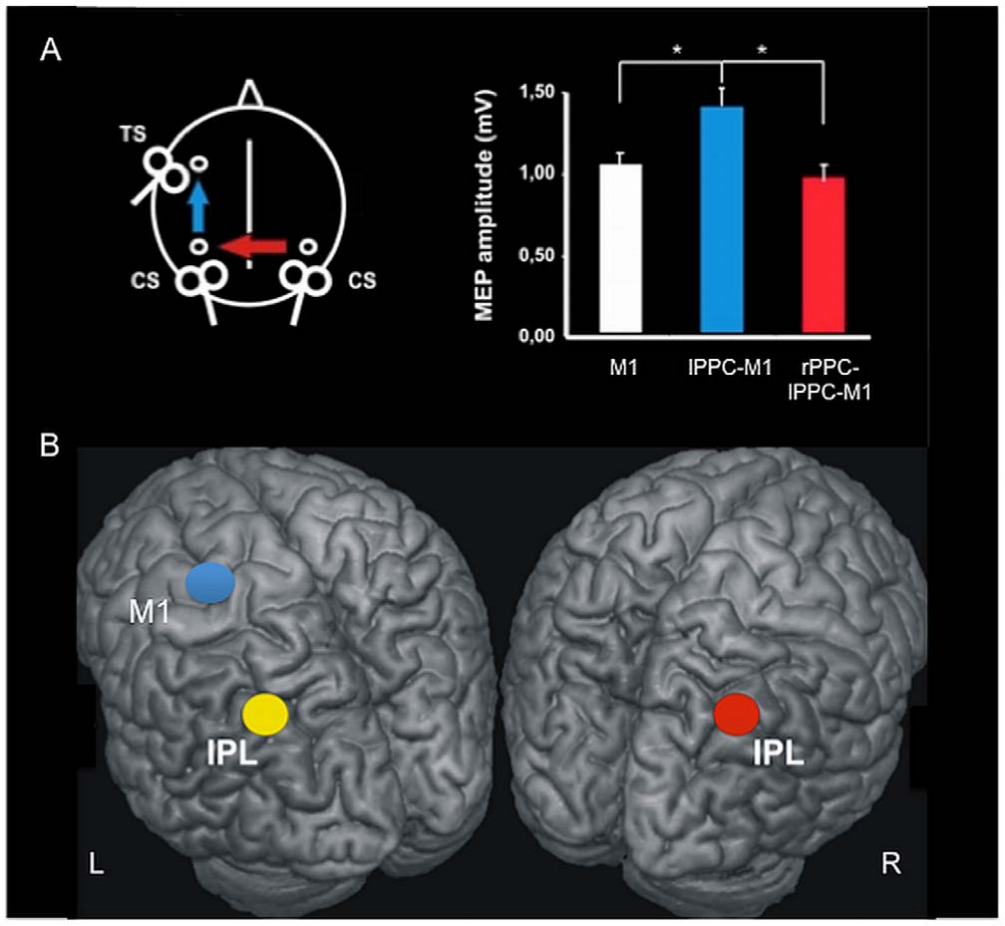
Cortico-cortical interactions explored with combined multifocal TMS and resting-state fMRI. A) Left posterior parietal cortex (PPC) TMS preceded left M1 TMS by 5 ms and induced increased MEP amplitude, indicating intra-hemispheric functional connectivity (blue arrow and blue column). When a third conditioning pulse was applied 10 ms earlier to contralateral PPC, the left intrahemispheric interaction was abolished, reflecting the activation of a transcallosal inhibitory pathway (red arrow and red column). B) Mean normalized MNI coordinates (x, y, z, mean±SD) of TMS PPC site were −48.2±4.8, –65.2±3.9, and 45.3±3.4 mm in the left hemisphere and 52.5±6.3, −60.2±4.7 in the right hemisphere. Mean MNI coordinates of left M1 were −30±3.3, −12±3.4, and 71±4.3.

A first test stimulus (TS) was applied over the hand motor areas of left M1 and was defined as the point in which stimulation evoked the largest motor evoked potentials (MEPs) from the contralateral first dorsal interosseous (FDI) muscle. Electromyographic (EMG) traces were recorded bilaterally from the FDI muscles using 9-mm diameter, Ag–AgCl surface cup electrodes. The active electrode was placed over the belly muscle, while the reference electrode was located over the metacarpophalangeal joint of the index finger. Responses were amplified using a Digitimer D360 amplifier (Digitimer Ltd, Welwyn Garden City, Hertfordshire, UK) through filters set at 20 Hz and 2 kHz with a sampling rate of 5 kHz, then recorded by a computer using SIGNAL software (Cambridge Electronic Devices, Cambridge, UK). The test stimulator for M1 was connected to a small custom-made figure-of-eight-shaped coil (external diameter 50 mm). The intensity of the TS was adjusted to evoke a MEP of approximately 1 mV peak to peak in the relaxed contralateral FDI muscles. To best activate the ipsilateral PPC-M1 facilitatory functional connection, a conditioning stimulus (CS1) applied over the PPC preceded the M1 TS by 5 ms, at an intensity of 90% of the ipsilateral resting motor threshold (RMT) (Fig. 1), which was defined as the lowest intensity that evoked five small responses (about 50 μV) in the contralateral FDI muscle, in a series of ten stimuli when the subject kept the FDI muscles relaxed in both hands [20]. In a previous study [21], we demonstrated that this connection is likely to involve a polysynaptic circuit that engages the ipsilateral premotor cortex (PMC). Although it might be argued that the interval that we used to test PPC–M1 interactions (ISI  = 5 ms) may be too short for an interposed synaptic connection in PMv, it should be noticed that the true latency of the interaction is complicated by the fact that a single suprathreshold TMS pulse to M1 evokes a series of I wave volleys in corticospinal neurones that can last 5 ms or more. Since all of these aspects contribute to the final amplitude of the MEP, inputs arriving as late as 9 ms after stimulation of PPC can affect the PPC–M1 interaction that we measure. Thus, an ISI of 5 ms may be sufficiently long to activate both, the direct and indirect (via PMC) circuit linking PPC to M1.

To investigate the functional inter-hemispheric connectivity between the PPC areas, a third TMS pulse (CS2) was applied over the contralateral PPC (PPC_CONTRA_) 10 ms before delivery of the PPC pulse ipsilateral to M1 (PPC_IPSI_), and therefore 15 ms before the M1 TS [16] (Fig. 1). This protocol is well known to activate an inhibitory transcallosal connection between the homologous PPC of the two hemispheres. We used a neuronavigation system (Softaxic, E.M.S., Bologna, Italy) to precisely position the coil (small custom-made figure-of-eight-shaped coil, external diameter 50 mm) over the PPC sites, using individual T1-weighted MRI volumes as anatomical reference; this technique has been previously described in detail elsewhere [15], [21]. The anatomical points used for stimulation were determined prior to the experiment, and were marked on the adherent plastic cap worn by the subject. The individual coordinates of each stimulation site were normalized *a posteriori* into the Montreal Neurological Institute (MNI) coordinate system and averaged [15], [21]. Within the PPC, the coil was positioned over the angular gyrus, close to the posterior part of the adjoining caudal intraparietal sulcus [22], [23]. The centre of the coils was positioned tangentially to the skull with the handle pointing downward and rotated medially by 15°. Each experimental session consisted of 45 trials. Three conditions were randomly intermingled: TS alone (MEP); PPC_IPSI_ + TS; PPC_CONTRA_ + PPC_IPSI_ + TS. Fifteen responses were collected for each condition. The inter-trial interval was 5 sec (±10%). Measurements were obtained for each individual trial. For correlation analyses, *intra-hemispheric PPC_IPSI_-M1 functional connectivity* was measured as the percentage of the mean peak-to-peak amplitude size of the MEP obtained by TS of M1 in isolation [15]. *Inter-hemispheric PPC_CONTRA-_PPC_IPSI_-M1 functional connectivity* was measured as the percentage of the mean peak-to-peak amplitude size of the MEP obtained by PPC_IPSI_ + TS of M1 [16]. MEPs were averaged across all trials and blocks for each condition and each subject.

### Statistical Analysis

A within-subject ANOVA was applied on mean MEPs amplitude with condition as main factor (TS alone (MEP), PPC_IPSI_ + TS, PPC_CONTRA_ + PPC_IPSI_ + TS) in order to verify the changes in the MEP amplitude following bifocal or trifocal TMS. *P* values <0.05 were considered as statistically significant. A significant main effect in the ANOVA was followed by post hoc paired *t* test analysis. The Greenhouse-Geisser correction for non-spherical data was used.

### Correlation between RS-fMRI and TMS Results

The across-subject correlation between fMRI and TMS data was assessed using a random-effect analysis in SPM8. Two models were set up for each of the 3 selected RSNs (DMN, DAN, and SMN). In the first model, intra-hemisperic PPC_IPSI_-M1 functional connectivity assessed by TMS (as defined above) was used as a regressor; in the second model the inter-hemispheric PPC_CONTRA -_PPC_IPSI_-M1 functional connectivity (as defined above) was used as a regressor. T contrasts were used to test the hypotheses of either positive or negative correlations.

*P*-values were considered as statistically significant if inferior to 0.05, after false discovery rate (FDR,[24]) correction at cluster level.

## Results

### Resting-state fMRI

**Figure 2:**
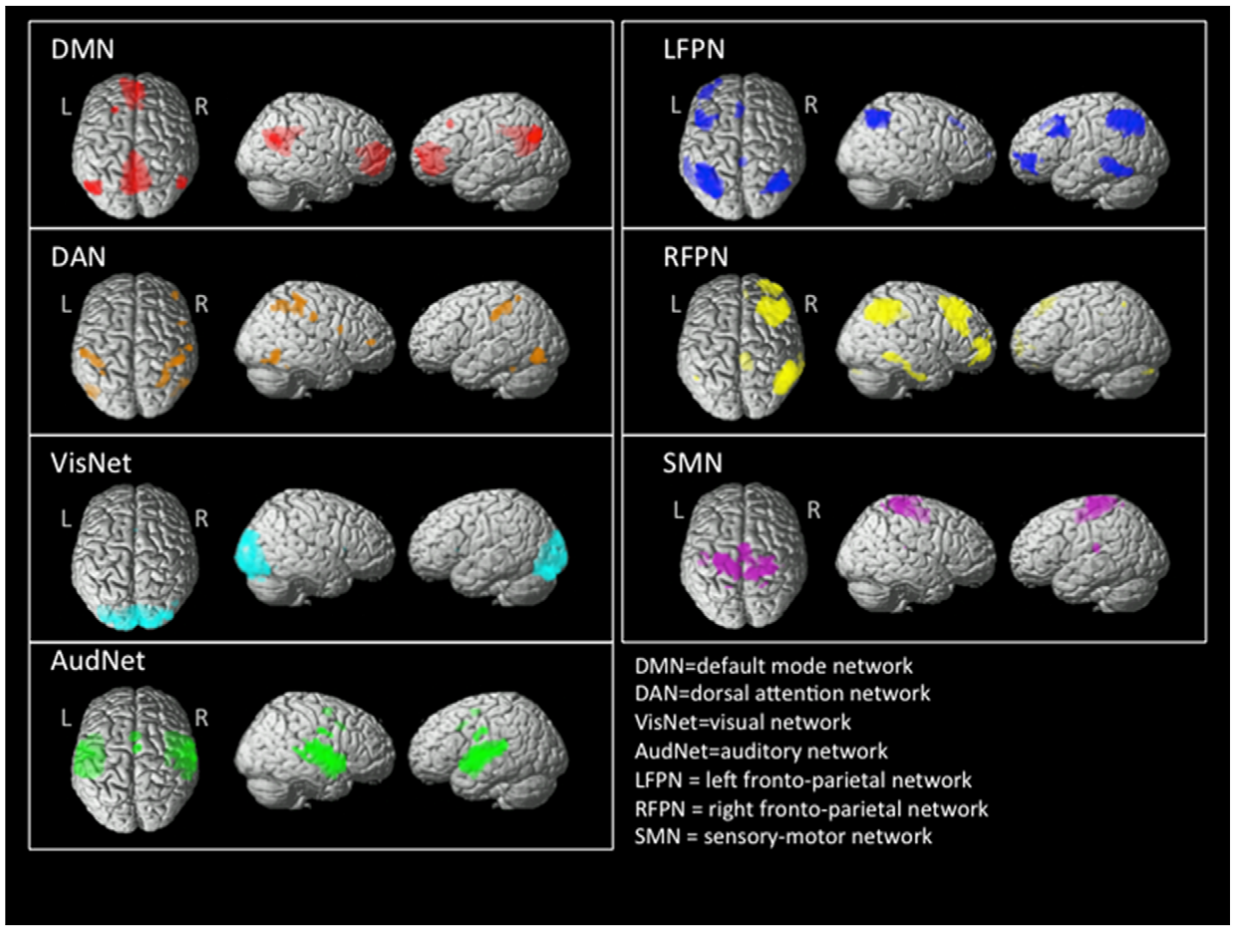
Three-dimensonal rendering of the resting state networks estimated by independent components analysis. The default mode network (DMN) has been linked to self-oriented mental activity; the dorsal-attention network (DAN) has been associated with goal-directed stimulus-response selection; The visual network (VisNet) has been associated with visual processing; the auditory network (AudNet) has been associated to processing of auditory stimuli; the left-lateralized fronto-parietal network (LFPN) and the right-lateralized fronto-parietal network (RFPN) have been associated to memory functions; the sensory-motor network (SMN) comprises primary sensory-motor areas and the supplementary motor area. For the correlation analysis, we selected the DMN, the DAN, and the SMN, based on anatomical considerations. See text for further details.

The RSNs identified among the components extract by ICA are shown in Fig. 2, and they include: the default mode network (DMN), the dorsal attention network (DAN), the visual network (VisNet), the auditory network (AudNet), the left fronto-parietal network (LFPN), the right fronto-parietal network (RFPN), and the sensory-motor network (SMN). These patterns are consistent with those previously published by other groups [8], [19].

### TMS

As expected, conditioning TMS applied to the left PPC produced an increase of MEP recorded by TMS applied over left M1 in isolation (ANOVA condition main effect F(2,38) = 16.04; p = 0.00001; t = −2.12; p = 0.03), which implies the activation of intra-hemispheric facilitatory functional connectivity [15] (Fig. 1A). This interaction is thought to involve a polysynaptic circuit that engages the ipsilateral premotor cortex (PMC) trough bundles of the superior longitudinal fasciculus (SLF) [21]. This same interaction was found to be completely abolished when another TMS pulse was applied over the contralateral PPC (trifocal TMS) (ANOVA condition main effect F(2,38) = 16.04; t = 2.81; p = 0.0007), thus indicating the activation of a transcallosal inhibitory pathway [16] (Fig. 1A). Notably, this inhibitory effect is known to be mediated by direct PPC-PPC transcallosal projections located in the posterior fourth of the corpus callosum, and not by a direct interhemispheric PPC-M1 pathway [16].

### Correlation between RS-fMRI and TMS Results

**Figure 3:**
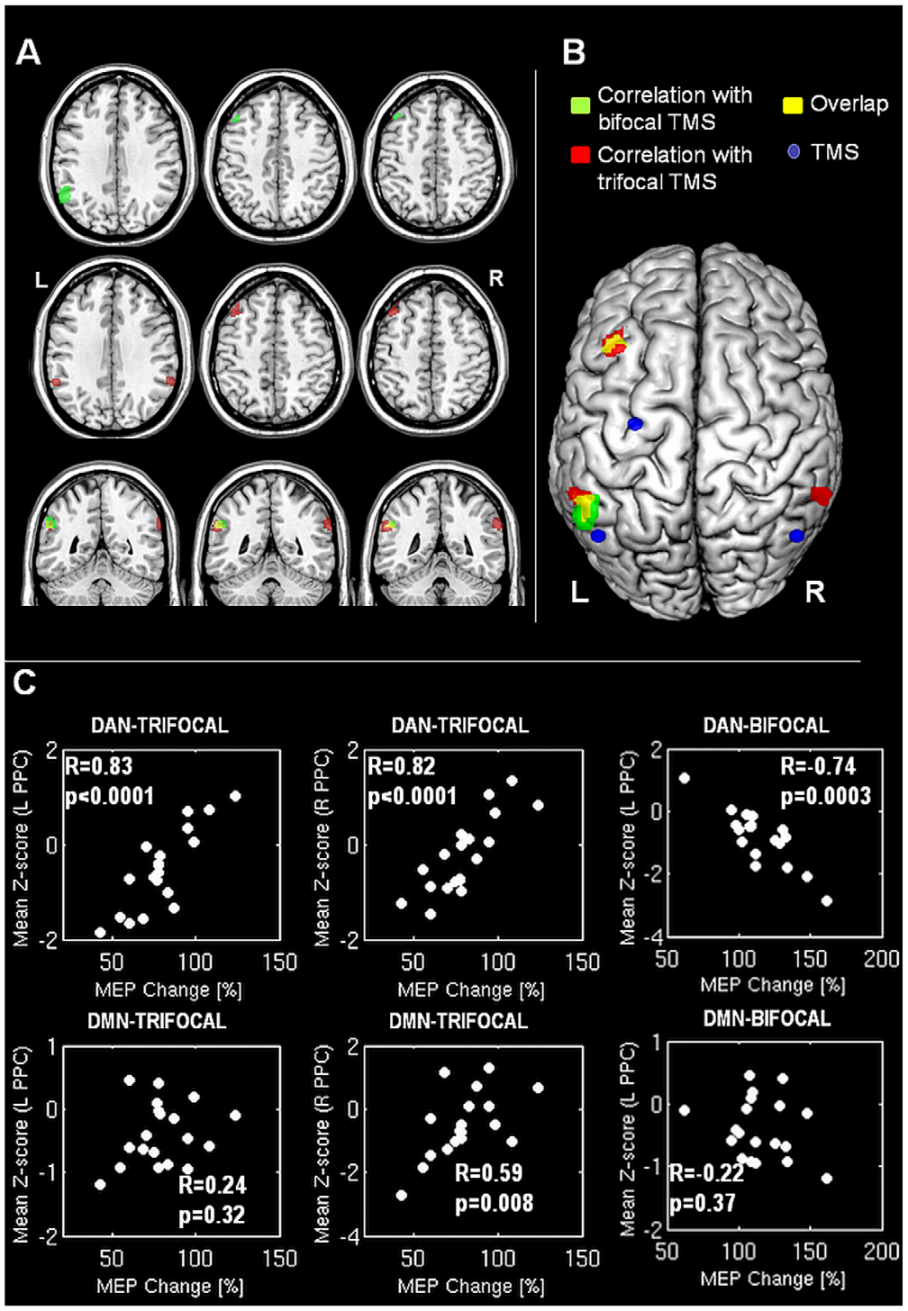
correlation between TMS and fMRI data. In panel A and B, areas of correlation between the dorsal attention network (DAN) and bifocal TMS are shown in green, while correlations between the DAN and trifocal TMS are shown in red. Overlapping regions are in yellow. Panel B also shows the sites of TMS stimulation (blue dots). The scatterplots reported in the upper row of panel C show the mean cluster Z-score (indicating the strength of functional connectivity estimated by resting state fMRI) against the percentage change in MEP for the 3 highlighted regions. In the lower raw of panel C, the corresponding mean Z-score relative to the default mode network (DMN) are also plotted against the percentage change in MEP (bottom row), but correlations were not significant. The directions of correlation (direct or inverse) are reversed in the two cases (bifocal and trifocal TMS) as expected due to the opposite (excitatory or inhibitory) nature of the underling connections. R and p values are estimated post-hoc using Pearson's correlation coefficient. See text for further details.

The integration of TMS and fMRI data revealed a remarkable correlation across subjects between functional connectivity (both intra- and inter-hemispheric) as measured by multifocal TMS, and the degree of correlation of spontaneous activity within the DAN [25], [26]. The left intra-hemispheric PPC-M1 functional connection activated by bifocal TMS stimulation correlated with functional connectivity within the DAN in two specific areas: one located over the left angular gyrus, close to the cortical site where left PPC TMS was applied (MNI coordinates [−56±4.3; −46±4.7; 30±6.2]); the other one within the left premotor cortex (MNI coordinates [−42±3.8; 24±4.7; 48±5.1]) (Fig. 3). The correlation with the angular gyrus survived after correction for multiple comparisons at cluster lever (p value after FDR correction  = 0.001). When the same analysis was extended to data obtained by trifocal TMS (inter-hemispheric connectivity), strong correlations were found in the same two areas (as for bifocal TMS), and in one additional region of the DAN, namely the contralateral PPC (MNI coordinates [62±5.1; −48±4.6; 30±6.2]) (Fig. 3). Also in this case, the spots over the left and right angular gyri were in proximity of the cortical sites where PPC TMS was applied (Fig 3B). All these correlations survived after FDR correction for multiple comparisons. No correlation was found between TMS data and functional connectivity within the other 2 RSNs explored. These results are summarised in Table 1. It is worth noting that the sign of the correlation was reversed for inter-hemispheric connectivity as opposed to intra-hemispheric connectivity. This is consistent with the hypothesis of an inhibitory circuit in the case of inter-hemispheric connectivity, and a facilitatory circuit in the case of intra-hemispheric connectivity.

**Table 1:** Results of the voxel-wise correlation between TMS and fMRI data.

Anatomical Location	MNI coordinates [x y z]	t-value	Cluster size [voxels]	p-value (FDR corrected)
Bifocal TMS vs Left supramarginal gyrus/angular gyrus	−54 −50 32	6.48	260	0.001
Bifocal TMS vs Left Middle frontal gyrus	−42 26 46	4.54	13	0.15
Trifocal TMS vs Left supramarginal gyrus/angular gyrus	−56 −46 30	6.95	149	0.042
Trifocal TMS vs Right supramarginal gyrus/angular gyrus	62 −48 30	6.66	112	0.042
Trifocal TMS vs Left middle frontal gyrus	−42 24 48	5.91	115	0.042

MNI = Montreal Neurological Institute; The p-values are corrected for multiple comparisons according to the false discovery rate (FDR) method.

In order to exclude the possibility that age and gender might have affected these results, we repeated the analysis, adding age and gender as covariates of no-interest. The results were consistent with those of the original analysis, although the statistical significance was reduced (remaining significant at p<0.1, FDR corrected at cluster level) due to the reduced number of degrees of freedom. A figure summarising the results of this analysis is provided on-line as Figure S1.

#### Post-Hoc Seed-based Analysis

In order to further explore our hypothesis, we performed some further correlation analysis between the fMRI and the TMS data. Every subject's functional data underwent an additional step of pre-processing, consisting of the removal of potential sources of spurious variance, including: global temporal drift using a 3^rd^ order polynomial fit, realignment parameters, and the signal averaged over whole brain voxels. The mean timeseries from the clusters found to be significantly associated with the DAN in the main analysis (left and right PPC and left premotor cortex) were extracted from the resulting images. The correlation coefficient between each pair of regions was estimated subject by subject using Pearson's formula. The resulting values (R_LPPC-RPPC_, R_LPPC-PM_, R_RPPC-PM_) were correlated across subject with the intra- and inter-hemispheric connectivity assessed using TMS.

No significant results were found. Correlation coefficients and p values are shown in Table 2.

**Table 2:** Results of post-hoc correlation performed between TMS measures of connectivity and fMRI time-courses.

	R_LPPC-RPPC_	R_LPPC-PM_	R_RPPC-PM_
Bifocal TMS	R = 0.24; P = 0.32	R = 0.25; P = 0.31	R = 0.29; P = 0.23
Trifocal TMS	R = −0.20; P = 0.42	R = −0.35; P = 0.14	R = −0.22; P = 0.36

Results of post-hoc correlation performed between TMS measures of connectivity and the average timeseries extracted for each of the regions of the dorsal attention network found to be significantly associated with TMS data using independent component analysis.

R_LPPC-RPPC_ = Pearson's correlation coefficient between left and right posterior parietal cortex;

R_LPPC-PM_ = Pearson's correlation coefficient between left posterior parietal cortex and left pre-motor cortex;

R_RPPC-PM_ = Pearson's correlation coefficient between right posterior parietal cortex and left pre-motor cortex.

## Discussion

Our data indicate that the correlation structure of hemodynamic signals recorded by resting-state fMRI is tightly related to the physiological interactions tested by methods based on non-invasive cortical stimulation. Crucially, we report here a strong overlap between the cortical areas selected for the TMS, and the resting-state areas of BOLD signal correlation within the DAN, but not with other RSNs, namely the DMN and SMN. It should be highlighted that the fMRI analysis was performed in a completely data-driven fashion, and did not require any *a priori* selection of target regions. The fact that no correlation was found between TMS measures, and connectivity within other RSNs such as the DMN, despite the anatomical proximity of its posterior parietal nodes with those of the DAN, confirms the reliability of our findings, which are highly specific to the DAN.

The DAN is supposed to be involved in voluntary visual attentional control trough a large-scale distributed network formed by the frontal, parietal and visual cortices [27]. Notably, we recently demonstrated that the PPC-M1 interactions tested here are likely to be involved in mechanisms of visual-spatial attention, as revealed by studies performed in healthy subjects [16] as well as in patients with hemispatial neglect [28]. Hence, the current TMS-fMRI approach provides a novel link between functional connections occurring at distant temporal time scales, thus revealing the degree of co-activation of slow metabolic changes, as detected by resting state fMRI in the range of seconds, to be associated to the physiological properties of fast pathways interacting in the range of few milliseconds.

Several previous studies have demonstrated that the fMRI RSNs signals correlate with EEG signals, suggesting that the different RSNs assemble through synchronization of electric activity as measured by EEG [7], [29]. Correlations between slow (<0.1 Hz) modulations of ongoing neuronal activity, as measured by local field potential (LFP) or multiunit activity (MUA), and fluctuations of the resting BOLD signal have been previously reported locally, near the microelectrode used for stimulation, as well as extending over other cortical regions [30]. Moreover, electrophysiological studies in both humans and animals point at slow fluctuations in high-frequency “gamma” local field potential (LFP) power (>30 Hz) as exhibiting spatial coherence over long timescales [31], with some evidence suggesting that this coherence is strongest between functionally related areas.

EEG/fMRI investigations, reporting an association between slow hemodynamic changes and faster electrical oscillations (up to 80 Hz), indicate a link between the ongoing connections that can be detected at different temporal scales. Notably, studies based on micro-state EEG provide evidence for a further association between slow resting state fMRI oscillations and neural activity in the scale of hundreds-milliseconds [9], [10]. Being uniquely characterized by a fixed spatial distribution of active neuronal generators with time varying intensity, a brain microstate might be considered as an electrophysiological fingerprint of specific neural processes occurring during relaxed wakefulness, such as mental imagery, abstract thought, sensory perception or memory retrieval [32] which are characterized by a specific occurrence with a mean duration of around 120 ms [9].

In this perspective, our findings reinforce the notion that the RSNs emerging from slow brain fluctuations are based on the neural activity of specific cortical substrates that operate at different time scales. These areas, belonging to the same network, might communicate with each other within different temporal scales, ranging from few seconds (timescale for resting-state fMRI oscillations) to few millisecond (timescale for TMS measurements).

A crucial issue that still remains to be clarified concerns how the activity of these connections actually contributes to specific mental activities [25], [33]. Previous findings showed that intrinsic low frequency fluctuations dramatically enhance the signal-to-noise ratio of task-based analyses [25] and that such intrinsic fluctuations are related to behavioural variability within participants [12]. These results suggest that a common mechanism governs the neural activity many brain regions' during rest as well as during task performance [34]. For instance, it would be of interest to investigate whether task-dependent changes of functional connectivity assessed with bifocal TMS [21], [35] could also be associated with individual variability of the correspondent RSNs.

In the present study, although the correlations of physiological interaction and fMRI functional connectivity were strongly significant, they were in the reversed direction than predicted: the lower the Z values of functional connectivity, the higher the facilitation induced by ipsilateral PPC stimulation in the bi-focal condition and the lower the inhibition induced by contralateral PPC stimulation in the tri-focal condition. With this regards, it has to be noted that these physiological interactions involve complex polysynaptic pathways mediated by inhibitory interneurons that could easily reverse the relationship between the physiological activity and the functional connectivity. Therefore it is difficult to provide a solid hypothesis to explain such apparent discrepancy.

It is also worth commenting on the inconsistency between the results of ICA and of the seed-based analyses. While strong correlations were found between TMS and DAN when using ICA decomposition, we were unable to demonstrate associations between TMS data and RS functional connectivity estimated by correlation analysis (see Table 2). Although in principle these 2 analysis methods should yield similar results, they process the time-series differently, which might explain the discrepancy we observed. It is important to observe that ICA may decompose the BOLD signal for a single region into the sum of several sources, corresponding to differing spatial components. Some of these sources might be assigned to noise components, which are not identified using seed-based analysis. The extent to which the BOLD signal is fragmented into several sources is also dependent on the number of components selected for ICA, which has to be decided a priori. A previous study [36] showed that despite a significant correspondence between the results provided by ICA and seed-based analysis this correspondence is not complete.

A limitation of the current study is that TMS and fMRI experiments were not simultaneously performed. It has indeed been argued that RSNs are non-stationary and may change within and between experimental sessions. In this view, the state of the subject might potentially alter the effects of TMS on EMG as well as on the connectivity of networks. For example, alertness, drowsiness, fatigue and so on could have considerable influence on the responsiveness as well as on the network activity and their spatial pattern.

In conclusion, we demonstrate that resting state fMRI may represent an effective method to investigate specific neurophysiological circuits and to quantify the degree of neuronal functional connectivity within them. We showed that combining multimodal TMS and resting state fMRI can effectively improve the characterization of the anatomo-functional properties of some crucial brain connections. Multifocal TMS, which is able to provide unique information concerning the inhibitory or facilitatory nature of a certain pathway with a sub-millisecond temporal precision, can be complemented by the high spatial resolution afforded by fMRI, thus allowing to detect more precisely the cortical nodes underlying functional connectivity. This approach could also strengthen the clinical use of resting state fMRI in several physiological and pathological conditions [37].

## Supporting Information

Figure S1 **Correlation between TMS and fMRI data, adjusting for age and gender.** In panel A and B, areas of correlation (p<0.1, FDR corrected for multiple comparisons at cluster level) between the dorsal attention network (DAN) and bifocal TMS are shown in green, while correlations between the DAN and trifocal TMS are shown in red. Overlapping regions are in yellow. Panel B also shows the sites of TMS stimulation (blue dots). The scatterplots reported in the upper row of panel C show the mean cluster Z-score (indicating the strength of functional connectivity estimated by resting state fMRI) against the percentage change in MEP for the 3 highlighted regions. In the lower raw of panel C, the corresponding mean Z-score relative to the default mode network (DMN) are also plotted against the percentage change in MEP (bottom row), but correlations were not significant.
(TIF)
