# Supplementary figures and images for: fMRI Resting Slow Fluctuations Correlate with the Activity of Fast Cortico-Cortical Physiological Connections
Source: PLoS One. 2012 Dec 20;7(12):e52660. doi: 10.1371/journal.pone.0052660 (PMC3527573; doi:10.1371/journal.pone.0052660)

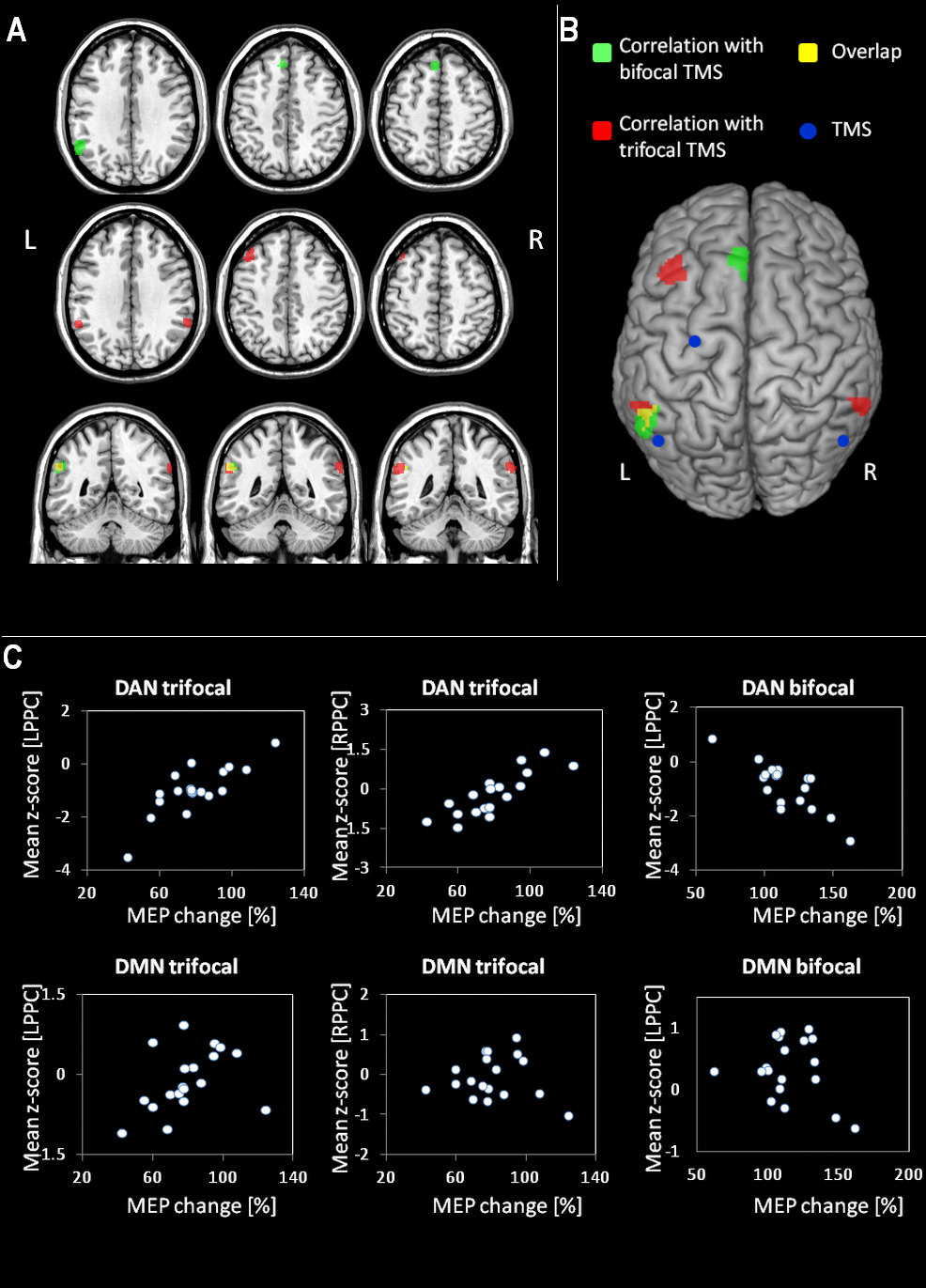

Supplement: Figure S1 — Correlation between TMS and fMRI data, adjusting for age and gender. In panel A and B, areas of correlation (p<0.1, FDR corrected for multiple comparisons at cluster level) between the dorsal attention network (DAN) and bifocal TMS are shown in green, while correlations between the DAN and trifocal TMS are shown in red. Overlapping regions are in yellow. Panel B also shows the sites of TMS stimulation (blue dots). The scatterplots reported in the upper row of panel C show the mean cluster Z-score (indicating the strength of functional connectivity estimated by resting state fMRI) against the percentage change in MEP for the 3 highlighted regions. In the lower raw of panel C, the corresponding mean Z-score relative to the default mode network (DMN) are also plotted against the percentage change in MEP (bottom row), but correlations were not significant. (TIF) [file pone.0052660.s001.tif]
